# *Lysimachia christinae* Hance as an anticancer agent against breast cancer cells

**DOI:** 10.1002/fsn3.1875

**Published:** 2020-09-10

**Authors:** Hyun A. Kim, Dong‐Sung Lee, Hwan Lee, Joomin Lee

**Affiliations:** ^1^ Department of Food and Nutrition Chosun University Gwangju Korea; ^2^ College of Pharmacy Chosun University Gwangju Korea

**Keywords:** apoptosis, breast cancer, epithelial–mesenchymal transition, *Lysimachia christinae* Hance

## Abstract

Breast cancer is the most common cancer in women, and metastasis is the leading cause of death in breast cancer patients. Although chemoprevention is widely employed to treat breast cancer, anticancer drugs can cause significant adverse effects. *Lysimachia christinae* Hance (LH) is a traditional Chinese medicinal plant with diverse therapeutic effects. However, its potential anticancer activity has not been fully investigated in breast cancers to date. Using high‐performance liquid chromatography–mass spectrometry, we found that the main constituent of LH extract (LHE) was rutin. Our results indicated that LHE or rutin markedly decreased the proliferation and viability of estrogen receptor (ER)‐positive MCF‐7 and ER‐negative HCC38 human breast cancer cells. LHE treatment induced morphological changes in apoptotic nuclei using 4′,6‐diamidino‐2‐phenylindole (DAPI) staining. Annexin V–fluorescein isothiocyanate (FITC) propidium iodide (PI) staining assay revealed that apoptosis significantly increased in both breast cancer cell types after LHE treatment. Additionally, the expression of poly (ADP‐ribose) polymerase (PARP), Bcl‐2, and phospho‐Akt decreased, while that of cleaved PARP and p53 increased, in both cell types. Furthermore, LHE treatment inhibited epithelial–mesenchymal transition (EMT). LHE treatment significantly upregulated E‐cadherin level in MCF‐7 and HCC38 cells, while vimentin level was downregulated in HCC38 cells. In addition, transwell and wound‐healing assays revealed that LHE or rutin inhibited breast cancer cell migration. Overall, these findings demonstrate that LHE is a promising therapeutic agent that acts by promoting apoptosis and reducing cell proliferation, EMT, and cell migration in ER‐positive and ER‐negative breast cancer cells.

## Graphical abstract

*Lysimachia christinae* Hance extract (LHE) effectively showed anticancer effects by inhibiting cancer cell proliferation and inducing apoptosis in estrogen receptor (ER)‐positive MCF‐7 and ER‐negative HCC38 human breast cancer cells. Moreover, LHE negatively regulated epithelial–mesenchymal transition (EMT) and decreased breast cancer cell migration.

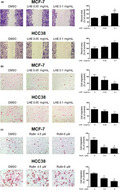

## 1 INTRODUCTION

Cancer is a disease that is a characterized by uncontrollable growth of cells and invasive behavior, which usually results in cancer progression and metastasis (Rajamanickam & Agarwal, 2008). Most breast cancer affects women, and 2.1 million diagnosed patients were recorded in 2018 (Ban & Godellas, 2014; Bray et al., 2018; Ghoncheh, Pournamdar, & Salehiniya, 2016). Current breast cancer treatments, such as early diagnosis, surgery of tumor, therapeutic radiation, immunotherapy, and cytotoxic chemotherapies, are largely successful (Waks & Winer, 2019). The selective estrogen receptor modulators (SERMs) are effective for hormone receptor‐positive breast cancers; however, high cost and side effects are often reported by breast cancer patients (Foulkes, Smith, & Reis‐Filho, 2010). Moreover, triple‐negative breast cancer (TNBC) patients without expressing estrogen and/or progesterone receptor (ER/PR) and human epidermal growth factor receptor 2 (HER2) had low five‐year survival and high recurrence rates after adjuvant chemotherapy (Al‐Mahmood, Sapiezynski, Garbuzenko, & Minko, 2018; Jordan, 2001; Sestak, 2014). Thus, it is necessary to find new active chemical compounds that are a less toxic approach for breast cancer treatment.

Plants and plant‐derived products have attracted increasing attention as inhibitors of the progression and development of cancers (Aung, Qu, Kortschak, & Adelson, 2017). Numerous drugs are from natural products or derived compounds and have made a significant contribution to anticancer, antimicrobial, and antioxidant activities (Balunas & Kinghorn, 2005; Tariq et al., 2017). *Lysimachia christinae* Hance (LH), a Chinese traditional medicine, belongs to the family *Primulaceae* and is widely used for the treatment of biliary calculus, hepatolithiasis, urinary calculi, damp jaundice, hepatobiliary lithiasis, and heat stranguria (13–16). Previous studies have identified the antidiuretic, antioxidant, and anti‐inflammatory effects of LH extract (LHE) (Gu, Zhang, & Nan, 1988; Tian, Yang, & Chen, 2008; Wang et al., 2018; Wu et al., 2018). LHE contains bioactive compounds such as flavonoids, phenols, and polysaccharides (Gu et al., 1988; Hong, Kim, & Lee, 2019; Wu et al., 2018). The medicinal properties of LHE are shown in literature, but the anticancer effects of LHE in breast cancers remain unknown. Therefore, we evaluated the compound in LHE or its major component rutin and their antiproliferation or antiviability effect on breast cancer cells (MCF‐7 and HCC38) as well as their anticancer effect on apoptosis and epithelial–mesenchymal transition (EMT), which has not been studied in the past.

## 2 MATERIALS AND METHODS

### 2.1 Preparation of *Lysimachia christinae* Hance extract (LHE)

The dry whole LH plant was purchased from a local market in Korea. The crude extract was obtained from the dry LH (100 g) using 1.5 L 80% ethanol. The crude ethanol extract was filtered using No. 1 Whatman filter paper. After vacuum evaporation, the extract from LH was obtained. The LHE was stored in a freezer at −20°C for further use.

### 2.2 High‐performance liquid chromatography (HPLC) analysis of LHE

The LH 80% ethanol extract was precisely quantified to 2 mg, dissolved in 1 ml of methanol and filtered to prepare an extract sample. The HPLC and Purospher HPLC column (Purospher^®^ STAR RP‐18 Endcapped, 4.6 × 250 mm, 5 μm, Merck, Darmstadt, Germany), consisting of a pump (1525 Binary HPLC Pump, Waters, Milford, MA, USA) and a PDA detector (996 Photodiode Array Detector, Waters), were connected and used for analysis. As mobile solvent systems, 0.1% acetic acid distilled water (A channel) and 0.1% acetic acid acetonitrile (B channel) were used. The slope profile proceeded as follows: 0–5 min, 5%–20% B linear; 5–85 min, 20%–30% B linear; 85–90 min, 30% B linear; 90–100 min, 30%–80% B linear; 100–105 min, 80%–100% B linear; 105–110 min, 100%‐5% B linear. The flow rate (1.0 ml/min) was maintained during the analysis. Next, 20 μl of LH 80% ethanol extract solution was injected at 2 mg/ml, and 20 μl of 1 mg/ml of rutin (≥94%, Sigma‐Aldrich, St. Louis, MO, USA) dissolved in methanol was injected as a standard sample. The detection wavelength was adjusted to 270 nm. The different concentrations of rutin (50, 100, 200 μg/ml) were subjected to quantitative analysis using the calibration graph.

### 2.3 Cell culture

Human breast cancer cell lines MCF‐7 (estrogen receptor positive) and HCC38 (estrogen receptor negative) were purchased from the American Type Culture Collection (Rockville, MD, USA) and maintained as suggested by the supplier.

### 2.4 Cell proliferation assay

MCF‐7 and HCC38 cells were then plated in a 96‐well plate at 3 × 10^3^ cells per well. LHE was dissolved in dimethyl sulfoxide (DMSO) to a final concentration of 0.4 g/ml and further diluted with cell culture medium. The cells were treated with 0, 0.1, 0.2, 0.4, and 0.8 mg/ml of LHE for 24 hr and 48 hr. The cell proliferation assay was performed as described by us previously (Lee, 2019).

### 2.5 Cell viability assay

Breast cancer cells (0.8 × 10^5^) in 12‐well plates were treated with LHE or rutin. After 24‐hr and 48‐hr treatment, cells were trypsinized and stained with trypan blue solution (Gibco, Thermo Fisher Scientific, Waltham, Massachusetts). Cells were then counted using a hemocytometer.

### 2.6 Flow cytometry assay of apoptosis

Apoptosis of MCF‐7 and HCC38 cells induced by LHE for 48 hr was analyzed using an Annexin V‐FITC Apoptosis Detection Kit (BD Biosciences, San Diego, CA, USA) according to the manufacturer's instructions. All the samples were analyzed using FACSCalibur (BD Biosciences).

### 2.7 Western blotting analysis

Following treatment with various concentrations of LHE, Western blotting analysis was performed as described by us previously (Lee, 2019) using anti‐β‐actin, anti‐PARP, anti‐Bcl‐2, and anti‐p53 (Santa Cruz, CA, USA) antibodies. E‐cadherin was from BD Biosciences. The antibody against vimentin was purchased from Sigma‐Aldrich. The antibody against cleaved poly (ADP‐ribose) polymerase (PARP), total Akt, and phospho‐Akt (Ser 473) were from Cell Signaling Technology (Danvers, MA, USA). The band density was quantified using ImageJ 1.41 software.

### 2.8 Confocal microscopy

A total of 1.5 × 10^5^ of each type of breast cancer cells were seeded overnight onto a 12‐well plate and then treated with LHE for 48 hr. After washing with PBS, the cells were fixed with 4% paraformaldehyde. After permeabilization with 0.1% Triton X‐100, the cells were stained with 4′,6‐diamidino‐2‐phenylindole (DAPI) and observed under a confocal microscope as previously described (Lee, 2019).

### 2.9 Cell migration assay

For wound‐healing assay, each type of breast cancer cells was allowed to grow into 80% confluent cells and then wounded with a pipette tip. The cells were incubated with medium containing 1% FBS and different concentrations of LHE. After 24 hr, the cells were fixed and stained with Giemsa stain solution. The area was analyzed using ImageJ 1.41 software. For transwell migration assay, serum‐free medium containing LHE or rutin with cells were placed into the upper wells of the chambers, and 5% FBS containing medium was added to the lower wells of the chambers. After 24 hr of incubation, the migrated cells were fixed with methanol and then stained with hematoxylin and eosin (H&E) stain (Sigma‐Aldrich).

### 2.10 Statistical analysis

All statistical analyses were performed using GraphPad Prism 6 software (San Diego, CA, USA). The *p* value <.05 was considered as a significant difference.

## 3 RESULTS

### 3.1 HPLC analysis of LHE

**FIGURE 1:**
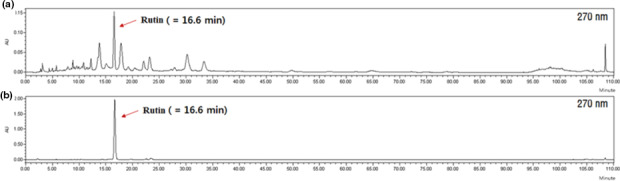
HPLC chromatogram of LHE (a) and rutin (b) as a standard compound. LHE, *Lysimachia christinae* Hance extract; HPLC, high‐performance liquid chromatography

We analyzed and confirmed the HPLC chromatogram of LHE and the standard compound rutin. Rutin is one of the major compounds in LHE (Sun et al., 2013). Data from the HPLC analysis of LHE and rutin were obtained in the form of chromatograms by monitoring detector responses at 270 nm. From Figure 1a, the retention time of the main peak in LHE was 16.6 min. Additionally, it was confirmed that the peak for rutin appeared at 16.6 min, and the LHE also showed a peak at the same retention time, 16.6 min (Figure 1). Additionally, we checked the content evaluation of rutin in LHE. The content values of rutin showed as 27.87 ± 3.32 mg/g (2.79 ± 0.33%) in the LHE sample. Therefore, this result suggested that LHE contained rutin as a major component.

### 3.2 LHE inhibits proliferation and viability in breast cancer cells

**FIGURE 2:**
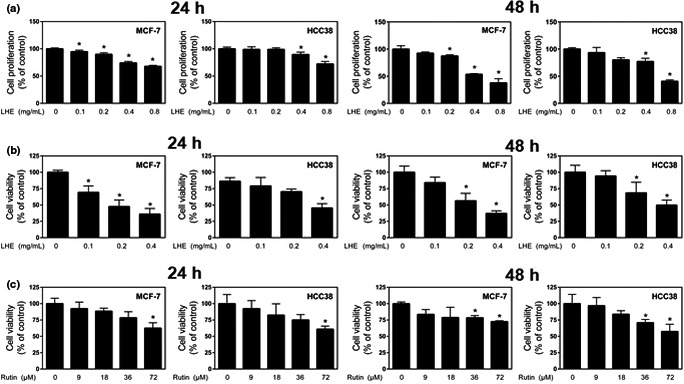
Effects of LHE on the proliferation or viability of MCF‐7 and HCC38 human breast cancer cells. Breast cancer cell lines were treated with LHE (0.1, 0.2, 0.4, 0.8 mg/ml) for 24 hr and 48 hr by MTS assay (a). Trypan blue exclusion staining assay was performed to determine cell viability using LHE (b) or rutin (c) for 24 hr and 48 hr in breast cancer cells. Data are expressed as the mean ± *SD* of at least three independent experiments. Significantly different (*p* < .05) compared with the ^*^DMSO control. LHE, *Lysimachia christinae* Hance extract; *SD*, standard deviation

Balance of cell proliferation and apoptosis is important for normal breast development, and its alteration is a crucial factor in cancer formation (Parton, Dowsett, & Smith, 2001). To investigate the effects of LHE on proliferation of breast cancer cells, cells were treated with the indicated concentrations (0–0.8 mg/ml) of LHE for 24 and 48 hr. Inner salt (MTS) assays using 3‐(4,5‐dimthylthiazol‐2‐yl)‐5‐(3‐carboxymethoxyphenyl)‐2‐(4‐sulfophenyl)‐2H‐tetrazolium showed that LHE induced a significantly higher inhibitory effect on MCF‐7 cells than on HCC38 cells (Figure 2a). Next, we examined the effect of LHE on cell viability using a trypan blue exclusion assay. A significant decrease in viability was observed with LHE treatment at 0.2 mg/ml and 0.4 mg/ml at 48 hr in both types of cancer cells (Figure 2b). In addition, we investigated the efficacy of rutin from LHE. The 0.2 mg/ml and 0.4 mg/ml LHE contained 9 μmol/L and 18 μmol/L rutin, respectively. Rutin treatment significantly inhibited breast cancer cell numbers from 36 μmol/L at 48 hr in breast cancer cells (Figure 2c).

### 3.3 LHE induces apoptosis in breast cancer cells

**FIGURE 3:**
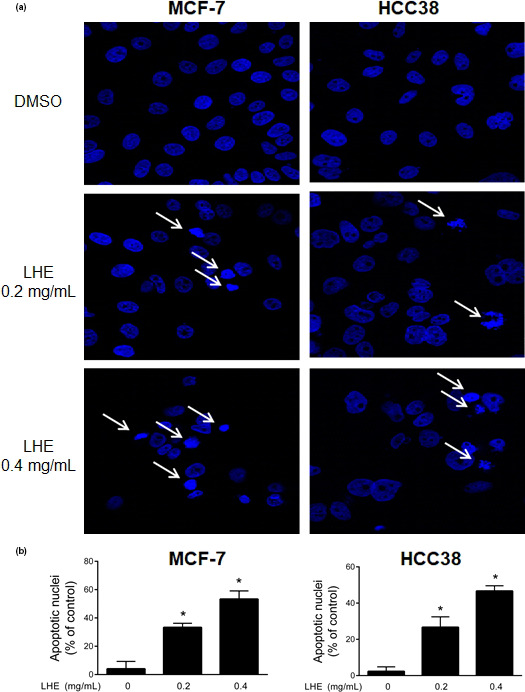
LHE‐induced apoptosis in breast cancer cells. (a) Apoptosis of MCF‐7 and HCC38 cells was increased by LHE extract. (b) The quantification of apoptotic nuclei. Data are expressed as the mean ± *SD* of at least three independent experiments. Significantly different (*p* < .05) compared with the ^*^DMSO control. LHE, *Lysimachia christinae* Hance extract; DAPI, 4′,6‐diamidino‐2‐phenylindole

**FIGURE 4:**
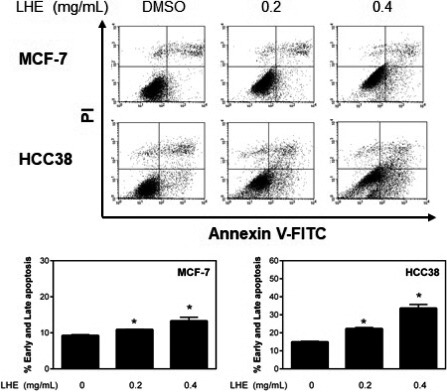
Effects of LHE on apoptosis in breast cancer cells. (a) The apoptotic cells were evaluated after annexin V‐FITC/PI staining using flow cytometry after LHE treatment for 48 hr. Quantification of the total apoptotic cell population (early + late) is shown. Data are expressed as the mean ± *SD* of at least three independent experiments. Significantly different (*p* < .05) compared with the ^*^DMSO control. LHE, *Lysimachia christinae* Hance extract; FITC/PI, fluorescein isothiocyanate/propidium iodide; *SD*, standard deviation

To determine the effect of LHE on apoptosis, MCF‐7 and HCC38 cells were treated with LHE for 48 hr. As shown in Figure 3, nuclei morphological changes and the appearance of apoptotic bodies were observed after LHE treatment. The levels of apoptosis induced by LHE in breast cells are shown in Figure 4. We found that LHE treatment showed a significant increase in early and late apoptosis rate in both types of cells. These data suggested that LHE may have an important pro‐apoptotic role in human breast cancer cells.

### 3.4 LHE activates apoptosis pathways in breast cancer cells

**FIGURE 5:**
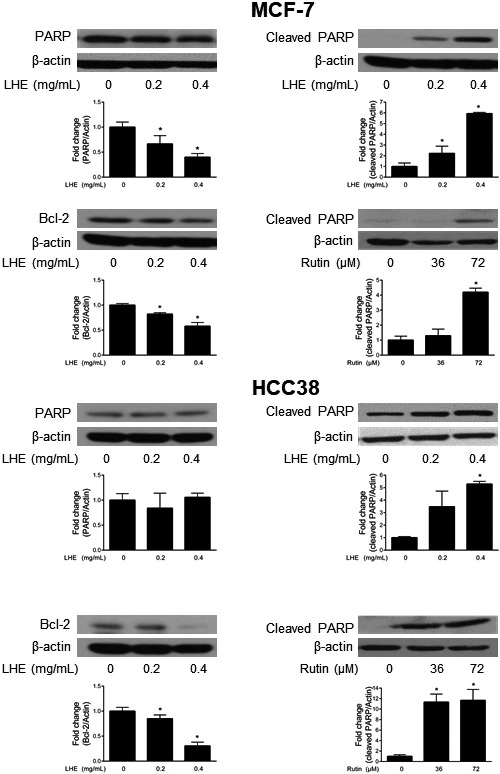
Western blotting analysis of PARP, cleaved PARP, and Bcl‐2 proteins in MCF‐7 and HCC38 cells treated with LHE or rutin for 48 hr. β‐Actin was used as the protein loading control. Data are expressed as the mean ± *SD* of at least three independent experiments. Significantly different (*p* < .05) compared with the ^*^DMSO control. PARP, poly (ADP‐ribose) polymerase; LHE, *Lysimachia christinae* Hance extract; *SD*, standard deviation

**FIGURE 6:**
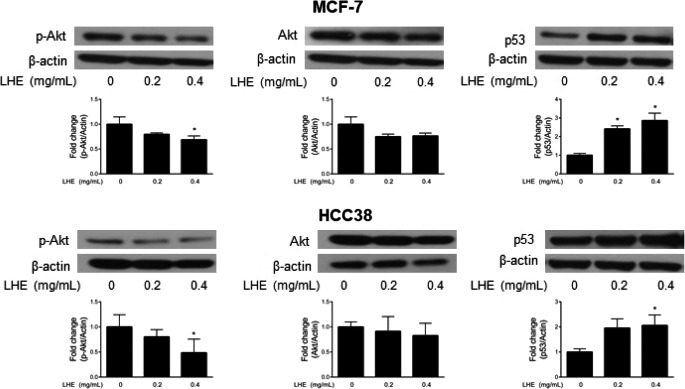
Western blotting analysis of p53, phospho‐Akt, and total Akt proteins in MCF‐7 and HCC38 cells treated with LHE for 48 hr. β‐Actin was used as the protein loading control. Data are expressed as the mean ± *SD* of at least three independent experiments. Significantly different (*p* < .05) compared with the ^*^DMSO control. LHE, *Lysimachia christinae* Hance extract; *SD*, standard deviation

To further investigate the mechanism of apoptosis after LHE or rutin treatment, we performed Western blot analysis in both breast cancer cell types. As shown in Figure 5, LHE treatment significantly decreased the expression of PARP, associated with increased cleaved PARP in MCF‐7 cells. Significant reduction in cleaved PARP was observed in HCC38 cells after 0.4 mg/ml LHE or rutin treatment. The level of Bcl‐2 significantly decreased with LHE treatment in both cell lines. Furthermore, LHE treatment showed increased expression of p53 in both MCF‐7 and HCC38 cells (Figure 6). Significant reduction in phosphorylated Akt expression was observed after 0.4 mg/ml LHE treatment without a change in the expression of Akt in breast cancer cells.

### 3.5 LHE modulates EMT in breast cancer cells

**FIGURE 7:**
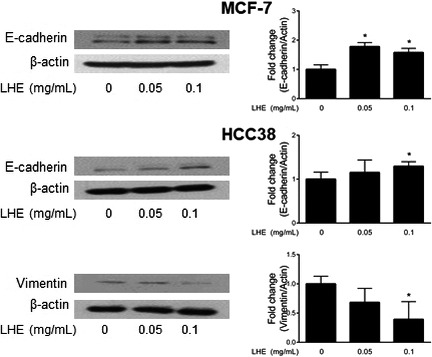
Effects of LHE on EMT‐related protein expression in breast cancer cells. Western blotting analysis of E‐cadherin and vimentin proteins in MCF‐7 and HCC38 cells treated with LHE for 24 hr. β‐Actin was used as the protein loading control. Data are expressed as the mean ± *SD* of at least three independent experiments. Significantly different (*p* < .05) compared with the ^*^DMSO control. LHE, *Lysimachia christinae* Hance extract; EMT, epithelial–mesenchymal transition; *SD*, standard deviation

To extend our knowledge of the function of LHE, we examined the protein expression of EMT in breast cancer cells. EMT plays a central role in invasion, migration, and development of metastases (Vincent‐Salomon & Thiery, 2003). Loss of E‐cadherin (epithelial marker) and acquisition of vimentin (mesenchymal one) are consistently observed during development and cancer EMT (Ashaie & Chowdhury, 2016; Zhang et al., 2014). Our results showed that LHE treatment (0.05 and 0.1 mg/ml) induced E‐cadherin expression in both cells (Figure 7). However, the expression of vimentin decreased with LHE treatment in HCC38 cells.

### 3.6 LHE inhibits migration of breast cancer cells

**FIGURE 8:**
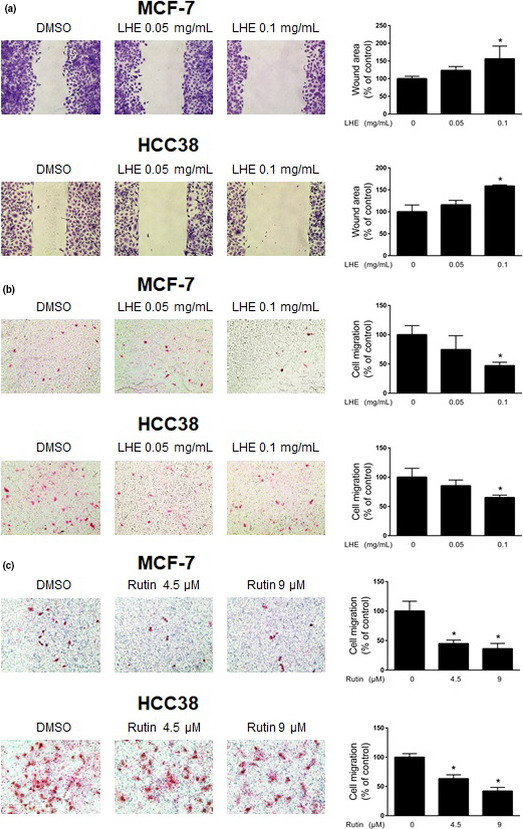
Effects of LHE or rutin on breast cancer cells migration in wound‐healing and transwell migration assay. (a) The confluent monolayers of MCF‐7 and HCC38 cells treated with LHE for 24 hr. Quantification of scratched area was performed using ImageJ software. (b) MCF‐7 and HCC38 cells in serum‐free medium with LHE or rutin were seeded into the transwell upper chamber. The lower chamber was filled with 5% FBS containing media. The cells were allowed to migrate for 24 hr. Migrating cells were stained with H&E solutions and counted. Data are expressed as the mean ± *SD* of at least three independent experiments. Significantly different (*p* < .05) compared with the ^*^DMSO control. H&E, hematoxylin and eosin; LHE, *Lysimachia christinae* Hance extract; *SD*, standard deviation

Epithelial to mesenchymal morphology in cancer cells drives migratory function and enhances the initiation of metastasis (Ashaie & Chowdhury, 2016). To investigate the effect of LHE or rutin on migration, breast cancer cells were exposed to LHE using a wound‐healing assay for 24 hr. LHE treatment decreased migratory capacity compared with a DMSO‐treated control in breast cancer cells (Figure 8a). Transwell migration also showed the same inhibitory migration pattern in MCF‐7 and HCC38 cells after LHE or rutin treatment (Figure 8b and c).

## 4 DISCUSSION

Cancer is a serious threat to human beings despite enhanced cancer management techniques including surgical operation and chemotherapy. Conventional treatments have often caused side effects, such as drug resistance and cancer metastasis. Therefore, new bioactive agents are needed for cancer prevention and treatment. Extensive research on safe and edible plants has revealed the activities of raw extracts or their chemical constituents (De Smet, 2002). Among them, *Lysimachia christinae* Hance (LH) has been studied for its protection against cholecystitis and cholagogic agents, and acute alcohol‐induced liver injury (Wang et al., 2012; Yang et al., 2011). However, little is known about LH’s anticancer effect in breast cancers. We, for the first time, investigated the antiproliferative, antiviability, pro‐apoptotic, and antimigratory effects of LHE or rutin as well as its inhibition of EMT in breast cancer cells.

It was reported that LHE contained flavonoids, phenols, and polysaccharides (Wu et al., 2018). We performed HPLC analysis and found rutin (quercetin 3‐O‐β‐d‐rutinoside) in LHE. Wu et al. also found that LHE contained flavonoid monomers including rutin (Wu et al., 2018). Rutin, a natural flavone derivative, has powerful antioxidant capacity by scavenging free radicals and chelating metals in vivo and in vitro (Javed et al., 2012; Kamalakkannan, Stanely, & Prince, 2006; Magalingam, Radhakrishnan, & Haleagrahara, 2013). Previous studies have shown that the components of LHE may contribute to its potent antioxidant effects by preventing hydrogen peroxide (H_2_O_2_)‐induced oxidative stress (Wu et al., 2018; Zhang, Liu, 2019). Our previous study also revealed that LHE has high antioxidant activities as tested by 2,2′‐azino‐bis‐3‐ethylbenzothiazoline‐6‐sulfonic acid (ABTS) and 2,2′‐diphenyl‐1‐picrylhydrazyl (DPPH) radical scavenging assays, ferric reducing antioxidant power (FRAP), and reducing power assay (Lee, 2019). Furthermore, rutin showed anticancer potential against breast (Iriti et al., 2017), colon (Nafees et al., 2018), and cervical cancers (Khan et al., 2020), and mesangial cells (Zhang, Wang, et al., 2019), suggesting that LHE has a number of bioactive compounds with antioxidant and anticancer activities. Further studies are needed on the other active compounds in LHE.

We then evaluated LHE against MCF‐7 and HCC38 breast cancer cell proliferation and viability. Uncontrolled cell proliferation is a characteristic of cancer, and many diverse factors such as mutations in proto‐oncogenes, tumor suppressors, or signaling pathways underly tumor development (Evan & Vousden, 2001; Sever & Brugge, 2015). The present study showed that LHE or rutin treatment resulted in loss of proliferation or viability in both cell types. In particular, estrogen receptor (ER)‐positive (+) MCF‐7 cells were more sensitive to LHE treatment compared to ER‐negative (−) HCC38 cells.

Apoptosis is the main form of programmed cell death, which plays important roles in cancer chemoprevention (Kitanaka & Kuchino, 1999). In this study, LHE treatment induced morphological changes, including condensation of chromatin and DNA fragmentation within the nucleus. Next, annexin V‐FITC/PI dual staining was conducted to determine whether LHE affects apoptosis in breast cancer cells. Treatment with LHE led to a significant increase in early (annexin‐positive/PI‐negative) and late (annexin‐positive/PI‐positive) apoptotic cells compared to DMSO control. Iriti et al. showed similar results where early apoptotic cells were approximately 15% and 18% after treatment with rutin, the major compound in LHE, in MDA‐MB‐231 and MCF‐7 cells, respectively (Iriti et al., 2017). Our results also showed that LHE treatment increased cleaved PARP and decreased antiapoptotic Bcl‐2 expression in both breast cancer cell types (Figure 5). The intrinsic mitochondrial apoptosis pathway plays a key role during cell death. This leads to caspase‐9/caspase‐3 activation, which cleaves PARP, leading to DNA fragmentation and apoptosis (Green & Kroemer, 1998). In addition, overexpressed Bcl‐2 and related antiapoptotic proteins have been reported to inhibit cell death and chemotherapy resistance (Del et al., 2003; Yoshino et al., 2006). Therefore, the antiapoptotic Bcl‐2 family can promote apoptosis and thus reduce side effects in cancer chemotherapy. It has been reported that the tumor suppressor gene p53 affects Bcl‐2 in apoptosis or cell cycle arrest (Tomita et al., 2006; Wiman & Zhivotovsky, 2017). The present study found that the expression of p53 increased after LHE treatment in both breast cancer cell types. Linjawi et al. showed that patients with Bcl‐2 expression correlated with estrogen and progesterone receptors and p53 mutations showed poor survival in early breast cancer (Linjawi, Kontogiannea, Halwani, Edwardes, & Meterissian, 2004).

Activated Akt, a well‐known survival factor, also leads to antiapoptotic activity, which results in prevention of the release of cytochrome *c* from the mitochondria (Whang, Yuan, Liu, Majumder, & Lewis, 2004). Akt is a serine–threonine kinase downstream of phosphatase and tensin homologue deleted on chromosome 10/phosphatidylinositol‐3,4,5‐triphosphate (PTEN/PI3K) (Datta, Brunet, & Greenberg, 1999; Link, Rosado, Fominaya, Thomas, & Carnero, 2005). PTEN acts as nuclear tumor suppressor, and the loss of PTEN increases genomic instability by causing increased Akt‐mediated sequestration of Chk1 resulting in a DNA‐damage response (Puc & Parsons, 2005). The activation of PI3K‐Akt downregulates p53 via the Mdm2 oncoprotein. Mdm2 can interact with p53 and promote its degradation by the proteasome (Mayo & Donner, 2002). Our data indicated that treatment with LHE resulted in significant reduction in phosphorylated Akt (Figure 6). These results suggest that LHE may have a potential role in apoptosis induction. More experiments are needed on the mitochondrial‐mediated apoptosis pathway.

EMT has been shown to be critical for metastasis, which changes the organization of epithelial cells into isolated, mesenchymal, and migratory phenotypes (Vincent‐Salomon & Thiery, 2003). During EMT, cells lose adherence junctions such as E‐cadherin and occludin, and gain mesenchymal markers such as vimentin, fibronectin, and N‐cadherin (Zhang et al., 2014). In breast cancer, partial or total loss of E‐cadherin increases cell migration and invasion (Ashaie & Chowdhury, 2016; Zhang et al., 2014). Meta‐analysis suggested that low E‐cadherin expression might predict the poor prognosis (Vincent‐Salomon & Thiery, 2003). Furthermore, activation of an EMT program correlates with increased metastatic potential and drug resistance in TNBC (Zhang et al., 2014). This study showed that the expression of E‐cadherin was upregulated, while the expression of vimentin was downregulated in LHE‐treated breast cancer cells. Furthermore, we showed that LHE or rutin treatment inhibited wound‐healing migration and transwell migration in both breast cancer cell types. These data suggest that LHE can reverse the EMT phenomenon and inhibit the migration of breast cancer cells.

In conclusion, our data demonstrate that LHE effectively showed anticancer effects by inhibiting cell proliferation and inducing apoptosis. Moreover, LHE negatively regulated EMT and decreased breast cancer cell migration in a range that does not cause cytotoxicity after LHE treatment. Therefore, LH may play a promising role in the treatment of breast cancers.

## CONFLICT OF INTEREST

The authors declare no conflicts of interest.
